# Psychological dynamics and adaptive processes of negative online social interactions among university students: a qualitative study in higher education digital environments

**DOI:** 10.3389/fpsyg.2026.1934182

**Published:** 2026-09-03

**Authors:** Wei Zheng, Haoran Chen, Jiayuan Gu, Xinwen Huan

**Affiliations:** 1 Business School, Changzhou University, Changzhou, China; 2 School of Government, East China University of Political Science and Law, Shanghai, China

**Keywords:** adaptive coping, cyberpsychology, negative online interaction, psychological dynamics, university students

## Abstract

**Objective:**

As digital platforms increasingly shape university students’ learning, social interactions, and identity construction, negative online social interactions have become important experiences in connected digital environments. Existing research has mainly examined specific negative online behaviors, such as cyberbullying and online exclusion, and their psychological consequences, while paying less attention to how students interpret and adapt to negative interactions embedded in everyday digital life. Drawing on a cyberpsychological perspective, this study explores the dynamic psychological processes and adaptation pathways underlying negative online social interactions among university students.

**Methods:**

A qualitative research design was adopted, using semi-structured interviews with 35 university students and 10 teachers and staff members. Student interviews constituted the primary analytical data, while teacher and staff interviews provided contextual insights into university digital interaction environments. Thematic analysis was conducted through iterative coding, researcher discussions, and reflexive analysis.

**Results:**

Four interconnected themes were identified: (1) high digital platform conductivity shapes negative online interactions, as platform immediacy, persistence, and visibility amplify the spread and retention of impacts; (2) continuous exposure transforms negative interactions into cumulative psychological burdens, involving emotional fluctuations, stress accumulation, and meaning reconstruction; (3) students regulate negative interaction impacts through progressive coping pathways, moving from immediate emotional responses to boundary adjustment and strategic behavioral adaptation; (4) resource integration facilitates adaptive adjustment in digital environments, with digital literacy, emotional regulation, social support, and psychological resilience jointly supporting psychological balance and sustained digital engagement. These findings indicate that negative online social interactions are not isolated events but dynamic processes shaped by technological features, psychological experiences, and social resources.

**Conclusion:**

This study extends the understanding of negative online social interactions beyond specific cyber harm incidents by conceptualizing them as ongoing psychological processes within digital social interactions. It reveals how university students experience interactional stress, develop coping pathways, and achieve adaptation in connected digital environments. The findings contribute to a process-oriented understanding of technology-mediated social interactions in cyberpsychology and provide practical implications for digital citizenship education, student mental health support, and healthier digital interaction environments.

## 1 Introduction

As digital technologies and social media platforms become increasingly integrated into the learning, daily lives, and social interactions of university students (Varsori and Pinho, 2026), technology-mediated digital interactions have emerged as crucial contexts for students to maintain relationships, express identity, and process social experiences (Caspi and Etgar, 2023). Notably, online interactions constitute a significant component of the everyday social lives of university students (Miconi et al., 2023). Within a continuously connected digital environment, the digital media landscape exerts a growing influence on individual psychological experiences (Zeng and Chen, 2026), behavioral decision-making, and the construction of social relationships. In recent years, the focus of research in online psychology theory and applied studies (Finnemann et al., 2026) has expanded from traditional online behaviors of university students to examining the dynamic changes in individual psychology and social behavior within digital social interactions. The emphasis is on investigating how technology-mediated environments continuously shape university students’ cognition, emotions, behaviors, and social relationships (Fortuna, 2023).

Digital platforms facilitate information sharing and social connectivity while simultaneously engendering a more complex interaction ecology (Gregori et al., 2024). Features such as platform openness and anonymity enable online interactions to transcend temporal and spatial constraints; yet they also render ordinary opinion expression (Gaisbauer et al., 2020), disagreements, value conflicts, and emotional displays (Armstrong-Carter et al., 2023) more prone to escalating into persistent negative online social interactions (Hutchins et al., 2021; Skogen et al., 2023). Aggressive commentary (Gordils and Jamieson, 2025), group cyberbullying, and various other forms of negative expression (Chen and Zhang, 2025) have become common experiences in university students’ daily digital lives (Miconi et al., 2023). Compared with traditional face-to-face conflicts, negative online interactions are characterized by persistent retention and rapid dissemination; their impact extends beyond immediate emotional responses (Dork et al., 2024) to continuously affect social evaluation, self-presentation, and other dimensions. Consequently, negative online interactions are not merely localized phenomena within digital platforms but represent a significant component of digital social interaction and serve as an essential entry point for understanding the psychological adaptation of university students in the digital age.

In recent years, research concerning university students’ digital social interactions and their psychological impacts has yielded considerable findings. However, existing scholarship offers limited explanations for how students navigate, comprehend, and respond to negative online social interaction processes within a persistently connected educational and social digital environment. On one hand, studies have investigated the effects of various negative online experiences on students’ mental well-being (Liu et al., 2017), exploring the relationships between different interaction types and psychological states such as depression (Mayer et al., 2025), anxiety (Anto et al., 2023; Keles et al., 2020), and self-esteem (Liu, 2025), to some extent elucidating the patterns of association between interaction experiences and students’ psychological states (Wu et al., 2022). On the other hand, research examining the protective roles of various factors in buffering negative online experiences has provided a crucial foundation for understanding the links between negative interactions and university students’ mental health (Shannon et al., 2022; Valkenburg et al., 2022). Furthermore, with the advancement of cyberpsychology, an increasing number of studies have begun to focus on the influence of digital factors on university students’ behaviors, including emotional fluctuations (He and Fan, 2025), stress experiences (Sundvik and Davis, 2023), and self-reflection (Yeo and Chu, 2025). Nevertheless, current research predominantly centers on the examination of variable relationships and the interpretation of mental health outcomes, largely addressing the correlational results of negative online interactions (Wu et al., 2022) while lacking in-depth explanations of the dynamic processes of perception, comprehension, regulation, and adjustment undertaken by university students in the digital social sphere. Current research exhibits several limitations: First, most studies focus on mental health outcomes such as anxiety and depression among university students (Vrabková and Vaňková, 2025), with less attention paid to the formation, accumulation, and evolution of psychological experiences during interaction processes. Second, a significant body of research employs cross-sectional surveys or structural modeling to analyze variable causal relationships (Al-Jayyousi et al., 2025; Mah et al., 2026), providing limited insight into the continuous psychological changes and behavioral adjustments of university students following negative online interactions. Third, existing studies often treat digital platforms as mere research contexts (Al-Hail et al., 2023; Wang et al., 2026), with scant focus on how the digital media environment itself, through processes such as open dissemination and anonymous expression, continuously influences university students’ psychological activities and social interactions. Fourth, current research remains predominantly quantitative (Monazam-Tabrizi et al., 2026; Xu et al., 2019), lacking process-oriented evidence regarding how university students comprehend negative interactions, ascribe meaning, and develop adaptive strategies.

Therefore, it is imperative to explore the formation processes of individual psychological dynamics within the social interactions of university students, facilitated by technological mediation, from the perspective of cyberpsychology (Finnemann et al., 2026). This endeavor aims to enrich the theoretical explanations within the research on digital social interaction. Cyberpsychology posits that digital platforms not only alter interpersonal information dissemination but also reshape multiple psychological processes, including social relationship formation and emotional expression (Fortuna, 2023). In a continuously connected digital society, the psychological activities of university students do not occur in isolation but are continuously constructed and adjusted through the synergistic influence of platform affordances, interaction contexts, and other multifactorial determinants. Therefore, rather than solely concentrating on the psychological consequences of negative online interactions, it is more crucial to examine how university students continuously perceive, interpret, and adjust their behavior within technology-mediated social interactions—that is, how their psychological dynamics evolve in tandem with interaction contexts (O'Keeffe and Clarke-Pearson, 2011). This research perspective, emphasizing the dynamic interplay of “interaction—psychology—behavior,” offers a more comprehensive understanding of the interactive experiences in the daily digital lives of university students and aligns more closely with current cyberpsychological research directions concerning technologically mediated social interaction.

Negative online interactions are highly contextualized, processual, and subject to individual differences (Luo and Gui, 2022). Relying solely on quantitative data makes it difficult to fully capture the complex and continuous psychological changes and behavioral adjustment processes individuals undergo during such interactions. Qualitative research, however, can provide an in-depth account of respondents’ authentic experiences within specific interaction contexts (Velardo and Elliott, 2021), revealing the intrinsic connections among psychological experiences, emotional shifts, meaning-making, and coping strategies. This approach facilitates a process-level understanding of how individuals continuously adapt to complex environments in digital social interactions. Accordingly, this study adopts a semi-structured interview method (Adeoye-Olatunde and Olenik, 2021) and systematically analyzes the real experiences of university students and teachers with negative online interactions. The aim is to construct a model of the psychological dynamics and adaptive processes involved in university students’ negative online interactions, thereby enriching cyberpsychology research with more process-oriented evidence. Consequently, this study focuses on investigating the following three research questions:

RQ1: *How do university students experience and interpret negative online social interactions in their everyday digital lives?*

RQ2: *How do their psychological responses evolve throughout these technology-mediated interactions?*

RQ3: *How do university students mobilize personal and social resources to adapt to such experiences?*

Drawing upon an in-depth qualitative analysis of the aforementioned issues, this research will meticulously investigate the psychological experiences and coping mechanisms employed by university students engaging in negative online social interactions (Colì et al., 2023). The study will concentrate on how individuals formulate psychological responses, adjust interactive behaviors, and mobilize resources such as digital literacy, emotion regulation, social support, and psychological resilience to achieve adaptation within a continuously connected digital environment. Comprehending the interactive processes of this specific demographic is paramount, as the university stage represents a critical transitional period for the development of academic identity, peer relationships, and digital citizenship practices. By examining how university students navigate negative online social interactions within their perpetually connected digital milieu, this study offers a contextualized understanding of cyberpsychology. Furthermore, it will enrich theoretical explanations within cyberpsychology concerning technology-mediated social interactions and furnish a theoretical foundation and practical implications for digital citizenship education in higher education, the construction of psychological support systems for university students, and the governance of healthy digital communities.

## 2 Literature review and theoretical framework

### 2.1 Negative online social interactions in digitally connected environments

As digital technologies and social media platforms are increasingly integrated into the learning, life, and social interactions of young people (Varsori and Pinho, 2026), online social interaction has become a crucial means for university students to establish relationships, express emotions, share perspectives, and shape their self-identity (Miconi et al., 2023). This study defines negative online social interaction as negative interpersonal experiences, such as psychological distress, social threat, or relational tension, encountered by individuals during digitally mediated communication processes. These experiences encompass hostile comments, interpersonal conflicts, misunderstandings, exclusion, and persistent negative feedback. This conceptualization extends beyond overtly aggressive behaviors like cyberbullying (Antoci et al., 2016) or online harassment (John et al., 2018; Kwan et al., 2020), focusing instead on how university students perceive, interpret, and adapt to negative experiences within their everyday digital interactions.

In a perpetually connected digital environment, online interaction has evolved into a novel form of social engagement shaped by both platform architecture and technological affordances (Ghazvineh, 2025). Digital platforms, characterized by their openness, immediacy, anonymity, algorithmic curation, and persistent information dissemination, have fundamentally altered individuals’ processes of information exchange, self-presentation, and social evaluation (Fortuna, 2023; Pathak et al., 2023; Rachadell, 2022). Concurrently, these technological features amplify the potential for misunderstandings, conflicts, and negative evaluations, leading to verbal aggression (Gordils and Jamieson, 2025), online exclusion (Hutchins et al., 2021; Skogen et al., 2023), miscommunicative exchanges (Chen and Zhang, 2025), and continuous negative feedback becoming common occurrences in the daily digital lives of university students.

Furthermore, digital media serve not merely as technological conduits for traditional social interactions but fundamentally reshape the formation and maintenance of social relationships among youth from a structural perspective. Nesi et al. (2018a,b) proposed the “Framework for Understanding Peer Relationship Transformation in Social Media Contexts,” positing that the technical characteristics of social media, such as visibility, persistence, accessibility, and feedback mechanisms, alter the processes of information exchange, self-presentation, and social evaluation among young individuals. This leads to relational dynamics in online interactions that differ from those in traditional face-to-face encounters. Research indicates that digital platforms not only influence positive peer connections but may also exacerbate risk processes such as social comparison, relational uncertainty, experiences of exclusion, and negative social evaluations (Nesi et al., 2018b). This theoretical perspective suggests that understanding negative online social interactions among university students necessitates an examination not only of specific negative behavioral types but also of how technical media characteristics modify the occurrence, persistence, and individual interpretation of interactions. Consequently, this study situates negative online social interactions within a context of continuous digital connectivity, focusing on how university students develop psychological experiences and adaptive strategies within technologically mediated social interaction.

Existing research has demonstrated that negative online interactions can precipitate anxiety (Anto et al., 2023), perceived stress (Wolfers and Utz, 2022), self-doubt (Liu, 2025), and emotional distress (Shaughnessy et al., 2017), subsequently impacting individuals’ social engagement, sense of belonging, and psychological safety in digital environments (Du, 2026; Rivas et al., 2020; Shannon et al., 2022; Valkenburg et al., 2022). However, current studies primarily concentrate on specific negative behavior types or psychological health outcomes, such as cyberbullying, online exclusion, and exposure to negative comments (Kwan et al., 2020; Skogen et al., 2023; Ai and von Mühlenen, 2025; John et al., 2018). There is limited explanation from an overall digital social interaction perspective regarding how individuals experience, interpret, and cope with negative interaction processes.

These interactions are particularly salient in higher education, as university students’ digital communication is embedded within intertwined spheres of course collaboration, peer relationships, and extracurricular activities. Therefore, this study does not focus on a single form of online harm but rather on the psychological dynamics and adaptive processes that emerge when university students encounter negative interactions within a continuously connected digital environment. Such interactions are typically integrated into students’ daily communication, expressions of interest, and maintenance of social relationships, with their psychological impact not being a discrete outcome but rather one that is continuously formed, accumulated, and adjusted through ongoing engagement. Individuals require emotional regulation, cognitive appraisal, relational negotiation, and behavioral adaptation to sustain psychological adaptation within digital environments (Han et al., 2023; Primack et al., 2017; Whaite et al., 2018). Consequently, understanding psychological dynamics of negative online social interactions can advance theoretical explanations in cyberpsychology about technologically mediated interactions and digital adaptation processes.

### 2.2 Negative online interactions from the perspective of stress and coping theory

Stress and coping theory (Folkman, 1984; Lazarus and Folkman, 2004; Wolf and Fisher, 2022) posits that individuals do not generate fixed responses directly to external stressful events; rather, they undergo a process of cognitive appraisal of the event’s meaning and an assessment of coping resources (Folkman, 1984; Lazarus and Folkman, 2004; Wolf and Fisher, 2022). Individuals’ understanding of the nature of the event, their evaluation of their own capabilities, and their perception of environmental resources collectively influence the subsequent selection of coping strategies. In recent years, a growing body of research has begun to conceptualize the online interaction environment as a distinct social context (Miconi et al., 2023). For university students, experiences such as negative comments (Ai and von Mühlenen, 2025), public criticism (Gordils and Jamieson, 2025), online misunderstandings (Chen and Zhang, 2025), and group arguments (Wu et al., 2022) are often perceived as stressful digital events. However, existing studies have predominantly focused on the psychological outcomes (Wolfers and Utz, 2022) and emotional experiences (Valkenburg et al., 2022) resulting from negative online interactions, with relatively limited attention to how individuals continuously appraise the situation, regulate emotions, and formulate coping strategies during the interaction process. Stress and coping theory provides a crucial lens for understanding this process, emphasizing the dynamic adaptation of individuals to stressful events rather than merely focusing on outcome variables. Therefore, applying this theory to the study of negative online interactions facilitates a deeper understanding of how university students interpret relevant experiences in digital environments and gradually develop distinct coping practices. Accordingly, this study examines respondents’ subjective experiences and coping processes in negative online interactions to delineate the specific trajectories through which their experiences unfold.

### 2.3 Digital resilience and adaptive resource mobilization in cyberpsychology

With the deepening development of the digital society, researchers have increasingly recognized that individuals are not passive victims when facing online risks (Han et al., 2024). Rather, through active learning, critical reflection, and the accumulation of experience, they continuously enhance their capacity to adapt to the digital environment (Zhu and Shen, 2020; Zhao et al., 2025). Digital resilience has gradually emerged as a core concept for explaining individuals’ digital adaptation processes. It is not only regarded as a foundational competency within contemporary comprehensive literacy frameworks (Zhu and Shen, 2020), but is also defined as an individual’s integrated capacity to actively respond to external changes and engage in self-regulation within the context of digital transformation (Zhao et al., 2025). The core of this concept lies in an individual’s sustained adjustment and dynamic recovery when confronting diverse challenges such as digital risks, negative social interactions, and online uncertainty. Relevant studies suggest that digital resilience does not manifest as the complete avoidance of risk (Crowder et al., 2026), but rather reflects a process of understanding, reflecting upon, and readapting to experiences following challenges (Sun et al., 2022; Vissenberg et al., 2022). For university students, this capacity depends on information judgment, emotional regulation, platform experience, and social support.

Digital literacy is also widely considered a significant component of digital resilience. A high level of digital literacy enables individuals to identify information sources, understand the rules of online interaction, and assess potential risks, thereby more effectively navigating complex online environments (Koltay, 2011). However, current research on digital literacy and digital resilience remains primarily at the level of quantitative measurement (Erdem et al., 2023; Park et al., 2021), lacking detailed descriptions of how they manifest and are experienced in specific negative online interaction contexts. Therefore, it is necessary to treat digital literacy and psychological resilience as important experiential resources that respondents may mobilize during interactions and to further explore their specific manifestations in coping practices.

### 2.4 Everyday digital interaction from the perspective of cyberpsychology

With the continuous development of digital society, cyberpsychology has gradually emerged as an important theoretical perspective for understanding individual psychology and social behavior in the digital age (Finnemann et al., 2026). Cyberpsychology emphasizes that digital media environments not only transform the ways in which information is transmitted between individuals but also reshape psychological processes such as the construction of social relationships, emotional expression, self-presentation, and social evaluation (Fortuna, 2023). In a persistently connected digital environment, technological features such as platform openness, anonymity, algorithmic recommendations, and rapid information dissemination render social interactions more immediate, sustained, and public, while also making individual psychological experiences more dependent on specific interaction contexts (Pathak et al., 2023; Rachadell, 2022). Consequently, cyberpsychology focuses more on how social interactions mediated by technology continuously shape individuals’ psychological experiences and behavioral adaptation.

From this perspective, negative online social interactions should not be understood merely as cyberbullying (John et al., 2018; Kwan et al., 2020), cyberviolence (Antoci et al., 2016), or specific online incidents (Winstone et al., 2023), but rather as a prevalent type of interaction experience embedded in the process of digital social interaction. For university students, academic communication, peer interaction, and public discussion increasingly take place on digital platforms. Negative interactions such as hostile comments, interpersonal conflict, miscommunication (Chen and Zhang, 2025), and online ostracism (Skogen et al., 2023) are typically embedded in everyday digital communication rather than occurring in isolation. Individuals’ psychological responses are not merely immediate reactions to single events; rather, they involve ongoing fluctuations in emotion, social evaluation, self-adjustment, and behavioral choices throughout the continuous interaction process, exhibiting distinct dynamic evolutionary characteristics.

**Figure 1:**
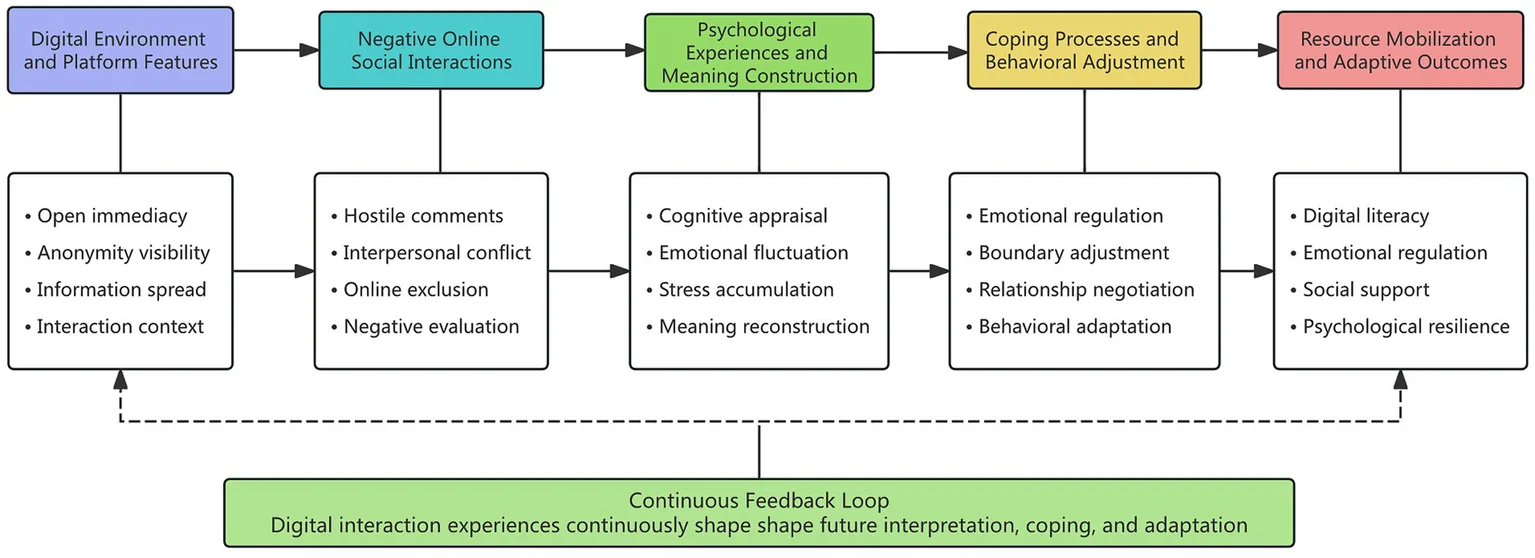
Theoretical framework. A five-step theoretical framework illustrating how digital environments shape negative online social interactions, psychological experiences, coping processes, and adaptive outcomes among university students.

This study examines the psychological experiences and adaptive processes associated with negative online social interactions among university students within the context of a continuously connected digital society. The research adopts a network psychology perspective, focusing on how individuals develop psychological experiences and regulate their interactive behaviors in technologically mediated social contexts, utilizing resources such as digital literacy for sustained adaptation. Prior research has predominantly focused on the relationship between negative online interactions and mental health outcomes. In contrast, this study prioritizes the formation and adaptation processes of psychological dynamics. From this theoretical standpoint, this research constructs a network psychology framework (Figure 1) for analyzing the psychological dynamics and adaptive processes of negative online social interactions among university students. Within this framework, digital platform characteristics shape the social context of negative online interactions, individuals form psychological experiences through cognitive appraisal and meaning construction, and subsequently achieve adaptation in the digital environment by employing coping strategies and mobilizing resources.

## 3 Methods

### 3.1 Research design

This study adopts a cyberpsychology perspective to understand technologically mediated social interaction, employing a qualitative research design (Colì et al., 2023). Qualitative social science research prioritizes the identification and interrelation of recurring themes within individual perceptions, as revealed through language and communication (Yang, 2024). Data were collected through semi-structured interviews using purposive maximum variation sampling (Liu et al., 2025), focusing on undergraduate students’ subjective experiences and coping processes during negative online interactions. Compared to research pathways focused on variable measurement, a qualitative approach is better for exploration of specific contexts to understand how individuals perceive, interpret, and respond to negative online interactions.

This study does not presuppose variable relationships or causal pathways. Instead, it inductively derives and interprets participants’ psychological experiences and coping practices based on their lived experiences. The interview guide was designed around the research questions and informed by relevant literature on negative online interactions, stress, and coping, and digital resilience. Key areas covered included the situational context of negative online interactions, emotional experiences, coping strategies, and the utilization of resources within digital environments. During interviews, the research team probed participants’ experiences, while maintaining consistency with thematic areas, to elicit richer data.

Acknowledging the differential sources of experience in negative online interactions between educators and students, distinct interview guides were developed for each group. Student interviews primarily focused on their direct experiences and coping practices, while teacher interviews concentrated on their observations and understanding of student educational management. By integrating the experiential narratives from both participant groups, this study aims to present the psychological experiences and coping processes of undergraduate students in negative online interactions from multiple perspectives. The online usage experiences collected in this study refer not to general internet use behaviors but specifically to participants’ technologically mediated social interaction experiences, including social media exchanges, online community participation, collaborative learning, and other forms of online interpersonal interaction.

### 3.2 Participants and sample

This study recruited a total of 45 participants: 35 undergraduate students (the primary research subjects) and 10 university teacher members with experience observing students’ digital interactions (contextual information providers). The students were recruited to explore negative online interaction experiences, psychological responses, and coping processes. The teachers were engaged to supplement understanding of students’ digital communication behaviors, interaction norms, and related support practices within educational settings, rather than to substitute for individual student experiences.

Recruitment employed a combination of purposive sampling (Palinkas et al., 2015) and voluntary participation. Students were recruited through announcements posted on campus digital channels, including WeChat groups, course groups, and student community platforms. The recruitment information introduced the study purpose, interview procedures, confidentiality measures, and participants’ right to withdraw. Interested students voluntarily contacted the research team and were subsequently screened to confirm whether their frequency of digital social interaction and prior experiences with negative online interactions met the established inclusion criteria. Teacher members were recruited through university teacher networks, with priority given to those who had experience observing or supporting students’ digital communication, course-related online interactions, or student organization activities. Their interviews were used to provide contextual insights into university digital interaction environments rather than to represent students’ personal online experiences. All participants reconfirmed their willingness and signed informed consent forms prior to formal interviews.

Inclusion criteria for students were: (1) consistent and frequent engagement in digital social interactions, including daily use of social media, instant messaging, or online communities; (2) prior experiences with negative online interactions, such as hostile comments, interpersonal conflicts, communication misunderstandings, online exclusion, or persistent negative evaluations. These criteria were designed to elicit participants’ psychological experiences and adaptation processes based on real-life digital interactions, rather than to require experiences involving extreme forms of online harm.

Teacher participants were required to have experience in student management, teaching, instructional guidance, or psychological support, and to have observed students’ engagement in course communications, class communities, campus digital communities, and digital citizenship education activities. Their interview data were solely intended to provide supplementary perspectives within educational contexts, aiding in the interpretation of the social environment and support systems surrounding student interactions, and were not used to infer private online behaviors.

Participants represented diverse disciplinary backgrounds across the humanities, social sciences, and natural sciences to enhance experiential variety. The research focused on undergraduate students’ understanding of and responses to negative interactions within continuously connected digital environments, encompassing various scenarios such as course-related communications, interest-based communities, and public social media platforms, thereby reflecting complex everyday experiences.

As a qualitative study, this research did not aim for statistical representativeness but rather emphasized an in-depth understanding of experiential processes within specific contexts. Comparative analysis was conducted continuously during data collection, and data collection was terminated upon reaching theoretical saturation (Guest et al., 2006), indicated by the point at which new interviews no longer yielded new core concepts. All participants signed informed consent forms, and research data were anonymized throughout the processes of organization, coding, and analysis.

### 3.3 Data collection

This study employed semi-structured in-depth interviews as the primary data collection method. An initial interview guide was developed based on a systematic review of the relevant literature and subsequently reviewed and refined by three experts specializing in cyberpsychology and higher education research. Prior to formal data collection, the research team conducted one pilot interview, and the question phrasing and interview procedure were further optimized in response to the feedback received, resulting in the final interview guide (Appendix 1). Interviews were conducted between March and April 2026 in Jiangsu Province, China, and were jointly administered by research team members Jiayuan Gu and Xinwen Huan. Each participant was interviewed once, with interviews lasting approximately 30–45 min. In consideration of participants’ schedules and practical constraints, 10 teacher members and 25 students were interviewed face-to-face; 5 students participated via video conferencing, and 5 students were interviewed using WeChat voice calls. All interviews were audio-recorded with the informed consent of the participants and were transcribed verbatim by the research team to produce textual data. To ensure comparability across different data sources, all interview types were analyzed using the same analytical framework and coding procedures.

During data analysis, the research team paid close attention to potential differences among data sources in narrative depth, degree of interaction, and modes of expression. A constant comparative analysis was employed to examine both commonalities and divergences across the different forms of data, thereby enhancing the credibility and robustness of the findings. The interview guide focused on participants’ specific experiences of negative online interactions, encompassing contextual factors of the interaction, event comprehension, emotional responses, coping practices, and the utilization of relevant resources. Separate but parallel interview guides were designed for students and teachers; however, the core topics remained consistent across both groups. The student interviews primarily addressed personal experiences and coping processes, whereas the teacher interviews emphasized their observations and understanding within the context of student educational management. All interviews were conducted with participants’ informed consent and were recorded in full with their permission. Following each interview, the recordings were transcribed in a timely manner, and all personally identifiable information was removed through anonymization to protect participants’ privacy and data security.

### 3.4 Data analysis

This study employed thematic analysis based on Braun and Clarke’s (2006) six-phase framework (Braun and Clarke, 2006), combined with a three-level coding approach commonly used in qualitative research, to systematically organize and interpret the interview data. The analytical process was not conducted as a linear sequence but developed iteratively through continuous comparison of interview materials, refinement of conceptual categories, and adjustment of theoretical interpretations. Detailed information regarding the coding procedures, category development, and the relationship between categories and final themes is provided in Section 4.2.

The analysis began with repeated readings of all interview transcripts to become immersed in participants’ core experiences and interaction contexts. Relevant meaning units related to the research questions were then identified line by line and assigned initial codes through open coding. Subsequently, conceptually related codes were compared, grouped, and integrated to develop higher-order categories. Finally, core themes were generated by revisiting the research questions and examining the relationships among themes. This process enabled the development of an integrated interpretation of university students’ negative online interaction experiences, including how emotional regulation shaped behavioral adjustment and how behavioral adjustments influenced social relationship negotiation.

To enhance the credibility and transparency of the analysis, several quality assurance strategies were adopted following established qualitative research criteria (Korstjens and Moser, 2018; Nowell et al., 2017). Two researchers independently conducted the initial coding and subsequently compared their interpretations. Discrepancies were discussed through repeated dialogue and negotiation, leading to refinement of category boundaries and theme definitions. The study did not employ quantitative reliability indicators, such as Cohen’s kappa, because such measures are inconsistent with the epistemological orientation of interpretive thematic analysis. Rather than assessing coding agreement as an outcome, the analysis emphasized researcher dialogue and reflexive engagement as means of developing a more nuanced and interpretive analytical framework. Throughout the process, the research team maintained reflexive records (Rogers, 2018) to critically examine how their own digital interaction experiences, educational backgrounds, and theoretical assumptions might influence data interpretation, thereby strengthening the contextual sensitivity and auditability of the findings.

During analysis, particular attention was given to how technology-mediated features shaped participants’ experiences of negative online interactions. Although conflicts, misunderstandings, and exclusion may also occur in offline settings, only experiences clearly influenced by digital characteristics were included in the core analysis. For example, when participants described experiences involving persistent information traces, rapid dissemination, reduced contextual cues, perceived anonymity, public visibility, or algorithmic exposure, these features were considered essential for understanding their psychological experiences. Accordingly, the coding process examined both the content of negative interactions and how digital affordances reshaped perceptions, interpretations, and coping, revealing distinct psychological dynamics in technology-mediated environments.

Furthermore, this study focused specifically on participants’ online social interaction experiences rather than general internet use. Interview data primarily concerned interactions occurring through instant messaging, social media platforms, online learning communities, and interest-based digital communities, ensuring alignment between the collected data and the research objectives. The analysis was not intended to establish causal relationships; instead, it aimed to understand, through participants’ lived experiences, how negative online interactions emerged, how they were interpreted, and how university students gradually developed strategies involving emotional regulation, behavioral adjustment, and resource mobilization within continuously connected digital environments. All analyses were conducted using anonymized data to ensure ethical research practice.

### 3.5 Researcher reflexivity

The research team’s reflexivity stems from their professional experience in higher education and student development. Researchers have extensive experience interacting with university students through face-to-face and technologically mediated educational engagements, including online course communications, academic advising, and student support activities. These experiences allow for a contextualized understanding of students’ digital interaction practices while necessitating ongoing reflexivity to avoid imposing researcher assumptions onto participant narratives. During analysis, the research team maintained reflective notes (Rogers, 2018) and repeatedly reviewed interview data to distinguish between participant experiences and researcher interpretations. Specifically, the interviews for this study were jointly conducted by two research team members experienced in online interaction practices and psychological support for university students; both received training in qualitative interview methodologies and possessed relevant research experience. Recognizing that researcher backgrounds may influence data collection and interpretation, the team maintained continuous reflexivity throughout the research process (Gesch-Karamanlidis, 2015) to enhance transparency and credibility. During interviews, open-ended and non-directive questioning was employed to encourage participants to articulate their experiences from their own perspectives, avoiding predetermined stances or value judgments. When discussions addressed negative online interaction experiences, the interviewers concurrently monitored participants’ emotional states and adjusted interview pacing and probing strategies as warranted. In the data analysis phase, the team performed iterative verification of coding outcomes and thematic extraction through independent coding, cross-review, and collective deliberation to mitigate the potential influence of personal experience and prior knowledge of interpretive findings. Researcher reflexivity and transparency reporting constitute essential strategies for strengthening the trustworthiness and auditability of qualitative research.

### 3.6 Informed consent and ethics approval

This study strictly adhered to the principles outlined in the Declaration of Helsinki (Sheather, 2024) and received approval from the Ethics Committee of Changzhou University (Approval No. 202606100001). Prior to the formal interviews, the research team communicated the study objectives, content, participation procedures, and privacy protection measures to all participants, from whom written informed consent was obtained. Participation was entirely voluntary, and individuals retained the right to withdraw at any time without consequence. To safeguard participant privacy, all interview data were anonymized; personally identifiable information was removed or replaced during transcription and analysis. The collected data are used exclusively for academic research purposes and are securely stored and managed by the research team.

## 4 Results

### 4.1 Participant characteristics

A total of 45 interviewees were recruited for this study (Tables 1, 2), comprising 35 university students and 10 teacher members with experience in observing student online interactions. Regarding gender distribution, among the student interviewees, 12 were male (34.3%), and 23 were female (65.7%); among the teacher interviewees, 3 were male (30.0%), and 7 were female (70.0%), indicating a female predominance in both groups. In terms of age, the student interviewees were mainly concentrated between 19 and 29 years old, while the teachers’ interviewees were primarily distributed across two age ranges—26–35 and 36–45 years—each accounting for 50.0%. Overall, the age gap between teachers and students offered distinct experiential perspectives.

**Table 1:** Summary of participant demographics (*N* = 45).

Category	Indicators	Student frequency	Student percentage	Teacher frequency	Teacher percentage
Gender	Male	12	34.3%	3	30%
	Female	23	65.7%	7	70%
Grade/target audience	Freshman	20	57.1%	8	80%
	Sophomore	5	14.3%	4	40%
	Junior	4	11.4%	2	20%
	Senior	6	17.1%	1	10%
Age	19–29	35	100%	0	—
	30–39	0	0%	5	50%
	40–49	0	0%	5	50%
	50–59	0	0%	0	—
Field of expertise	Natural sciences	14	40%	5	50%
	Humanities and social sciences	21	60%	5	50%
Years of work experience	8+ years	33	94%	9	90%
	5–8 years	1	3%	1	10%
	3–5 years	1	3%	—	—

**Table 2:** Respondent details and codes (*N* = 45).

Identity	Gender	Educational background	Major	Professional title	Code
Teacher	Male	Master	Chinese language and literature	Lecturer	TT01
Teacher	Female	Master	Agronomy	Lecturer	TT02
Teacher	Female	Bachelor	Tourism management	Lecturer	TT03
Teacher	Male	Doctorate	Communication	Associate Professor	TT04
Teacher	Female	Master	Criminal law	Teaching assistant	TT05
Teacher	Female	Master	Ideological and political education	Lecturer	TT06
Teacher	Female	Doctorate	Design	Lecturer	TT07
Teacher	Female	Doctorate	Business administration	Associate professor	TT08
Teacher	Male	Doctorate	Safety engineering	Professor	TT09
Teacher	Female	Doctorate	Safety engineering	Professor	TT10
Student	Female	Bachelor	Accounting	—	ST01
Student	Female	Bachelor	Chinese painting	—	ST02
Student	Male	Bachelor	Petroleum engineering	—	ST03
Student	Female	Bachelor	Accounting	—	ST04
Student	Male	Bachelor	Light chemical engineering	—	ST05
Student	Female	Bachelor	Ideological and political education	—	ST06
Student	Male	Bachelor	Electrical engineering and automation	—	ST07
Student	Female	Bachelor	Chinese language and literature	—	ST08
Student	Female	Bachelor	Clinical pharmacy	—	ST09
Student	Female	Bachelor	Physical education	—	ST10
Student	Female	Bachelor	Logistics management	—	ST11
Student	Female	Bachelor	Biopharmaceutical engineering	—	ST12
Student	Female	Bachelor	Medical Information engineering	—	ST13
Student	Female	Bachelor	Biopharmaceutical engineering	—	ST14
Student	Male	Bachelor	Logistics service and management	—	ST15
Student	Male	Bachelor	Automobile inspection and repair	—	ST16
Student	Male	Bachelor	Electrical engineering and automation	—	ST17
Student	Female	Bachelor	Chinese language and literature (teacher training)	—	ST18
Student	Male	Bachelor	Petroleum engineering	—	ST19
Student	Male	Bachelor	Electrical engineering and automation	—	ST20
Student	Male	Bachelor	Electronic information engineering	—	ST21
Student	Male	Bachelor	Physics	—	ST22
Student	Male	Bachelor	Electronic information engineering	—	ST23
Student	Male	Bachelor	Physics	—	ST24
Student	Male	Bachelor	Optoelectronic information science and engineering	—	ST25
Student	Male	Bachelor	Electronic information engineering	—	ST26
Student	Male	Bachelor	Electronic information engineering	—	ST27
Student	Male	Bachelor	Communication engineering	—	ST28
Student	Female	Bachelor	Physics	—	ST29
Student	Female	Bachelor	Optoelectronic information science and engineering	—	ST30
Student	Male	Bachelor	Electronic information engineering	—	ST31
Student	Male	Bachelor	Optoelectronic information science and engineering	—	ST32
Student	Male	Bachelor	Electronic information engineering	—	ST33
Student	Male	Bachelor	Human resources and management	—	ST34
Student	Female	Bachelor	Law	—	ST35

With respect to the academic year and teaching targets, the student sample was predominantly composed of first-year undergraduates (57.1%, *n* = 20), followed by sophomores (14.3%, *n* = 5), juniors (11.4%, *n* = 4), and seniors (17.1%, *n* = 6). Similarly, the teacher interviewees primarily taught first-year students (80.0%), followed by sophomores (40.0%), with juniors and seniors constituting 20.0 and 10.0% of their teaching targets, respectively. This distribution suggests that first-year students represent a significant participant group in university online interactions and are also a key focus for teachers in terms of daily online interaction guidance and observation.

In terms of disciplinary background, among student interviewees, 60.0% (*n* = 21) were from the humanities and social sciences, and 40.0% (*n* = 14) from the natural sciences; among teacher interviewees, the distribution was equal between the humanities and social sciences and the natural sciences (50.0% each, *n* = 5 each). Overall, the sample covered diverse academic fields, facilitating the representation of varied online interaction experiences.

Regarding internet usage history, the vast majority of interviewees had extensive experience with internet access and use. Among student interviewees, 94.3% (*n* = 33) had been internet users for over 8 years; among teacher interviewees, the corresponding proportion was 90.0% (*n* = 9). The remaining interviewees had internet histories concentrated between three and 8 years. Overall, the interviewees generally possessed rich internet usage experience and online interaction history, providing a substantial experiential basis for recalling and describing specific contexts, psychological experiences, and coping practices in negative online interactions.

During the interviews, student interviewees primarily recounted their own experiences of negative online interaction contexts, psychological experiences, and coping strategies, while teachers’ interviewees offered supplementary perspectives based on student educational management, online behavior observation, and practical experience. Diverse interviewees in identity, age, and discipline constituted the empirical source, yielding contextual data to understand university students’ psychological experiences and coping with negative online interactions.

### 4.2 Three-level coding process and theme extraction

Drawing on the three-level coding framework of grounded theory (Brink et al., 2006; Holt et al., 2022), this study systematically analyzed 45 interview transcripts to extract psychological experiences and coping processes related to negative online interactions from respondents’ experiential narratives. The coding process followed a progressive analytical path of open coding, axial coding, and selective coding, employing constant comparison and iterative induction to gradually integrate and distill concrete experiences into conceptual categories and core themes.

#### 4.2.1 Open coding

During the open coding phase, the research team first produced verbatim transcripts of all 45 interview recordings and conducted a meticulous sentence-by-sentence and paragraph-by-paragraph reading and analysis of the textual data to identify meaning units relevant to psychological experiences and coping processes in negative online interactions. To remain as close as possible to the respondents’ authentic experiences, the coding process preserved original expressions where feasible and used the respondents’ own language as a key basis for concept naming, thereby minimizing the influence of researcher preconceptions on analytical outcomes. Through repeated comparison and preliminary induction, the team identified approximately 180 initial concepts. Subsequently, by integrating semantically similar concepts and refining or eliminating low-frequency and redundant concepts, 42 first-level codes were ultimately established. These codes encompass multiple dimensions, including the situational contexts of negative online interactions, individuals’ emotional experiences, coping practices during interactions, and variations in experiential expression across different individuals, thus preliminarily revealing the multidimensional experiential features of this phenomenon. The representative raw expressions, keyword extractions, and summarized viewpoints generated during the open coding phase (Table 3).

**Table 3:** Open coding of psychological experience and coping strategies in negative online interactions.

Verbatim statements (interviewee responses)	Key terms	Derived insights
“Comment sections readily escalate into arguments; a single remark can spark controversy.”	Commentary conflict	Interactions readily escalate into conflict
“Minor offline conflicts are often amplified when transferred to online spaces.”	Conflict amplification	Online environments amplify interactional tension
“Individuals tend to express themselves more directly online, sometimes without fully considering the consequences.”	Direct expression	Reduced expressive constraints
“A single post can rapidly reach a large audience.”	Rapid dissemination	High information diffusivity
“Social media feeds and group chats can transform into forums for debate and even conflict.”	Multi-scenario interaction	Routinization of interactional contexts
“Viewing others’ comments can induce anxiety, with users unsure how to respond.”	Tension	Pronounced immediate emotional reactions
“Users may repeatedly check comments, with each review exacerbating distress.”	Repeated checking	Emotions exhibit accumulative properties
“A single comment can cause prolonged emotional distress.”	Sustained impact	Strong emotional persistence
“The emotional experience of online interactions differs markedly from face-to-face encounters.”	Contextual differences	Emotion is context-dependent
“Concerns about social evaluation and others’ perceptions are heightened.”	Evaluation anxiety	Significant social evaluative pressure
“Initial reactions often involve direct rebuttal or confrontation.”	Response	Tendency toward immediate responses
“Over time, individuals may refrain from engaging, deeming responses unnecessary.”	Abandonment of response	Shifts in coping strategies
“Some users opt to avoid comment sections entirely.”	Avoidance	Proactive reduction of contact
“Seeking peer support through private conversations is a common coping strategy.”	Seeking support	Intervention of external support
“Gradually, users learn to disengage from such discussions.”	Disengagement from interaction	Evolution of coping pathways
“More rationally oriented individuals prioritize verifying information accuracy before responding.”	Information appraisal	Differences in digital literacy
“A deliberate approach involves pausing to calm oneself before replying.”	Delayed response	Emotion regulation capacity
“Assuming leadership roles, such as class monitor, provides greater experience in managing these situations.”	Experiential accumulation	Social experience influences coping
“Extraverted individuals are more inclined to express themselves assertively.”	Personality differences	Individual traits affect expression
“Social support from friends significantly alleviates the negative impact”.	Social support	Buffering effect of resources

#### 4.2.2 Axial coding

Building upon the open coding phase, the research team further categorized and integrated first-level codes sharing similar attributes, common orientations, or inherent interrelations, thereby forming more abstract conceptual categories. This phase focused on the connections among codes and their manifestations within specific contexts, employing constant comparative analysis to delineate the experiential features reflected in the codes and their interrelationships. The research team systematically synthesized the initial codes across dimensions such as the contexts of negative online interactions, psychological experiences, coping practices, and individual resources (Table 4). For instance, codes such as “more direct online expression” and “frequent emotional expression” were subsumed under “openness of the expressive environment”; codes like “tension,” “anxiety,” “unease,” and “emotional dysregulation” were grouped under “emotional fluctuation”; and codes including “blocking others,” “withdrawing from discussions,” and “reducing social exposure” were consolidated under “avoidance and withdrawal”. Through repeated comparison and categorization of relevant codes, the research team progressively developed a set of conceptual categories that captured common experiential features across participants. Following multiple rounds of discussion and revision, a total of 19 s-level codes were established. These codes systematically represent the experiential characteristics of university students’ contextual perceptions, psychological experiences, coping pathways, and resource mobilization in negative online interactions, thereby laying the groundwork for the subsequent extraction of core themes.

**Table 4:** Axial coding of psychological experience and coping mechanisms in negative online interactions.

First code	Second code
Online expressions are more direct; emotional expressions are frequent.	Openness of the expressive environment
Arguments in comment sections; opposing viewpoints; escalation of conflict.	Overt conflict and diffusion of interactions
Dormitory conflicts extend online; real-world relationships affect online interactions.	Online-offline interweaving;
Diverse sources of information; difficulty distinguishing truth from falsehood.	Information complexity
Social media becomes a space for emotional catharsis.	Platformization of emotional expression
Tension; anxiety; unease; loss of emotional control.	Emotional fluctuations
Concern about others’ opinions; fear of being judged.	Perceived social evaluation pressure
Continuous attention to comments; repeated rumination over interactions.	Accumulated psychological burden
Worry that expression affects one’s image.	Self-presentation pressure
Emotional responses; direct rebuttals; catharsis.	Immediate responsiveness
Ignoring, adjusting expression, reducing participation.	Adaptive strategies
Blocking others, withdrawing from discussions, and reducing social exposure.	Avoidance and withdrawal
Seeking help from peers, teachers, or family members.	Utilization of external support
Shifting from participation to avoidance or observation.	Strategic shifts
Information judgment ability; contextual understanding ability.	Digital literacy performance
Emotional regulation; delayed response.	Emotion regulation capacity
High participation in activities; rich experience.	Variance in social experience
Extroversion/introversion; differences in expressive tendencies.	Individual trait differences
Interpersonal networks; support channels.	Social support resources

#### 4.2.3 Selective coding

Following axial coding, this study further integrated second-level codes around the core research question—“Psychological Experiences and Coping Processes in Negative Online Interactions”—to extract core themes with theoretical explanatory power. This phase emphasized systematic coding and theoretical completeness, ultimately yielding four third-level themes focusing on situational characteristics, psychological experiences, coping pathways, and individual resource perception (Table 5).

**Table 5:** Selective coding: psychological experiences and coping in negative online interactions.

First code	Second code	Core theme
Online expressions are more direct; emotional expressions are frequent.	Openness of the expressive environment	Theme 1: digital platform conductivity shapes the emergence of negative online social interactions
Arguments in comment sections; opposing viewpoints; escalation of conflict.	Overt conflict and diffusion of interactions	
Dormitory conflicts extend online; real-world relationships affect online interactions.	Online-offline interweaving;	
Diverse sources of information; difficulty distinguishing truth from falsehood.	Information complexity	
Social media becomes a space for emotional catharsis.	Platformization of emotional expression	
Tension; anxiety; unease; loss of emotional control.	Emotional Fluctuations	Theme 2: continued exposure drives negative interactions to transform into cumulative psychological burden
Concern about others’ opinions; fear of being judged.	Perceived Social Evaluation Pressure	
Continuous attention to comments; repeated rumination over interactions.	Accumulated Psychological Burden	
Worry that expression affects one’s image.	Self-Presentation Pressure	
Emotional responses; direct rebuttals; catharsis.	Immediate Responsiveness	Theme 3: students regain a sense of control over digital interactions through a progressive coping pathway
Ignoring, adjusting expression, and reducing participation.	Adaptive Strategies	
Blocking others, withdrawing from discussions, and reducing social exposure.	Avoidance and Withdrawal	
Seeking help from peers, teachers, or family members.	Utilization of External Support	
Shifting from participation to avoidance or observation.	Strategic Shifts	
Information judgment ability; contextual understanding ability.	Digital literacy performance	Theme 4: resource mobilization facilitates adaptive adjustment in digital environments
Emotional regulation; delayed response.	Emotion regulation capacity	
High participation in activities; rich experience.	Variance in social experience	
Extroversion/introversion; differences in expressive tendencies.	Individual trait differences	
Interpersonal networks; support channels.	Social support resources	

#### 4.2.4 Assessment of theoretical saturation

To examine the sufficiency and stability of the analytical results, the research team continuously monitored whether newly collected interview materials generated novel conceptual categories or themes during the coding process. Specifically, after establishing the core coding framework, the team analyzed subsequent interview transcripts and iteratively compared the new content with the existing coding scheme to determine the emergence of any significant new concepts or experiential categories. The results indicate that, as coding progressed, the content of newly added interview materials could largely be subsumed into the existing coding framework. In later interviews, respondents primarily supplemented, enriched, and validated pre-existing concepts without introducing substantively distinct new themes or core categories. Moreover, key concepts within each theme were repeatedly mentioned across different respondent groups, suggesting a high degree of consistency and stability in the reported experiences (Mizumoto, 2023). As the interview sample continued to expand, no additional core themes were identified, and the existing thematic structure adequately captured the respondents’ principal experiences of psychological responses and coping strategies in negative online interactions. Accordingly, the study concludes that the developed coding system has reached a satisfactory level of theoretical saturation, thereby providing a reliable empirical foundation for subsequent analysis (Table 6).

**Table 6:** Saturation of codes on psychological experiences and coping in negative online interactions.

Core themes	Code count	Student references	Teacher references	Novel concept emergence	Saturation
Psychological harm from negative interactions	28	34/35	10/10	Stabilization occurs following nine occurrences	★★★★★
Individual difference factors	22	30/35	8/10	Stabilization occurs following six occurrences	★★★★
evolution of coping strategies	18	28/35	5/10	Stabilization occurs following 12 occurrences	★★★★★
Effectiveness of support systems	15	25/35	7/10	Stabilization occurs following seven occurrences	★★★★
Gender differences in characteristics	12	15/35	6/10	Stabilization occurs following five occurrences	★★★
Formation of defensive systems	16	22/35	4/10	Stabilization occurs following 8 occurrences	★★★★
Demand for digital literacy	14	18/35	8/10	Stabilization occurs following six occurrences	★★★

### 4.3 Core findings

**Figure 2:**
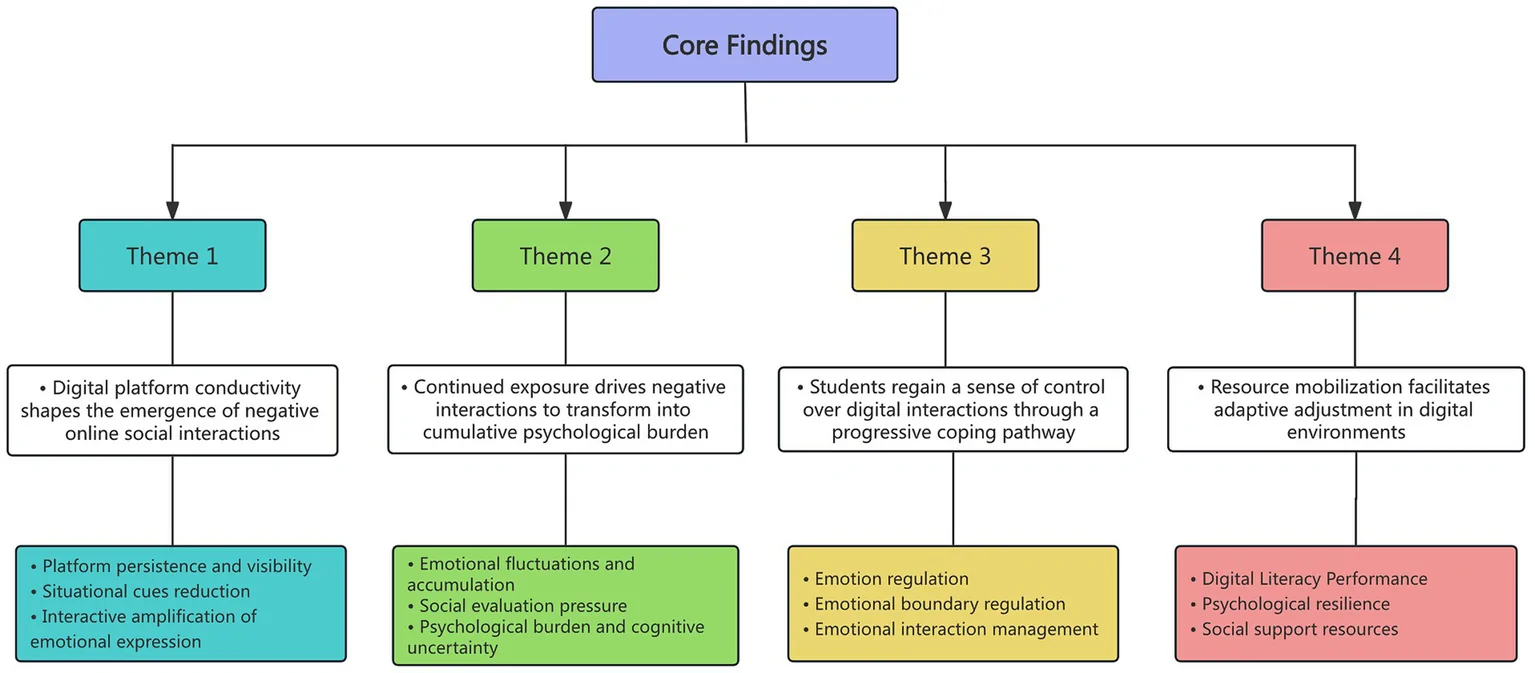
Core themes. Thematic structure of psychological experiences, coping processes, and resource mobilization in university students’ negative online social interactions.

This study is structured around three interrelated research questions: Firstly, how do university students experience and interpret negative online social interactions in their everyday digital lives (RQ1), Secondly, how do their psychological responses evolve throughout these technology-mediated interactions (RQ2), Thirdly, how do university students mobilize personal and social resources to adapt to such experiences (RQ3). Based on thematic analysis, this research does not treat negative online social interactions as isolated incidents. Instead, it elucidates a continuous dynamic process, from the formation of digital interaction conditions and the accumulation of psychological burdens to the adjustment of coping strategies and the adaptation through resource integration. Consequently, the following four themes should not be regarded as independent categories, but rather as constituting the psychological dynamic trajectory of university students within technologically mediated social interactions. Through systematic coding and constant comparative analysis of interview data, this study ultimately distilled four interconnected core themes: Theme 1: digital platform conductivity shapes the emergence of negative online social interactions; Theme 2: continued exposure drives negative interactions to transform into cumulative psychological burden; Theme 3: students regain a sense of control over digital interactions through a progressive coping pathway; Theme 4: resource mobilization facilitates adaptive adjustment in digital environments (Figure 2).

#### 4.3.1 Digital platform conductivity shapes the emergence of negative online social interactions

##### 4.3.1.1 Platform persistence and visibility

The interaction scenarios in which university students engage are widely distributed across platforms such as WeChat Moments, group chats, and social media comment sections. These spaces are characterized by rapid information dissemination and low expressive constraints, constituting a highly open communication environment (ST14, ST12, ST10). These examples illustrate that university students’ daily interactions are no longer confined to formal or pre-defined discussion spaces but are embedded in interfaces such as Moments, group chats, and comment sections—deeply integrated into everyday life. Overall, their expressive contexts exhibit a clear trend toward routinization and diversification.

“*One particularly memorable instance was when I shared on WeChat Moments that I had received a national scholarship*” (ST14);

“*The most unforgettable time was in the class QQ group, when I accidentally forwarded a message to the group*” (ST12);

“*The most memorable occasion was on Weibo, when I left a few comments encouraging table tennis player Wang Chuqin*” (ST10).

##### 4.3.1.2 Situational cues reduction

Online information sources are diverse and often indiscernible in terms of veracity. The online dispute mentioned in ST04’s interview typifies, from its initial context, the profound impact of “information complexity” on individual online interaction experiences. In this case, the information sources exhibit notable diversity and opposition: on the one hand, the respondent had a long-term interest in the young singer, forming a positive perception, and posted benevolent content that combined factual statements with emotional expression on social media; on the other hand, the opposing group relied on fragmented, unverified negative information to offer unsubstantiated criticisms of the singer. Both types of information coexisted on Douyin, a high-traffic, highly interactive social platform, thereby forming a direct information conflict field. In assessing information veracity, ST04 intended to clarify facts and believed their post was inoffensive. However, due to information complexity, the audience did not fairly receive this truthful and benevolent information; instead, soon after posting, it rapidly drew collective malicious interpretation and attack.

“*Last year, I experienced an online dispute on a social platform. I have always liked a young singer and would often watch his performances on social media, occasionally posting complimentary updates—simply sharing my admiration. One day, I came across a topic about him on Douyin, with many people discussing it. I saw someone slandering him baselessly, spreading unfounded negative remarks, which made me very upset. I only wanted to clarify the facts, so I carefully composed a passage listing his small efforts, without any offensive language—just hoping to prevent misunderstandings about him. Yet shortly after posting that comment, I was met with a wave of vicious attacks from a large group. Some people indiscriminately accused me of blind fandom, mocking me with harsh words. Others combed through my previous daily posts, making derogatory remarks about my photos and preferences, and even sent abusive messages to my private inbox. I merely wanted to defend someone I admire, but was instantly engulfed by overwhelming hostility”* (ST04)

##### 4.3.1.3 Interactive amplification of emotional expression

In the accounts of students ST33, ST27, and ST19, social media emerges as a significant arena for university students to vent emotions during negative online interactions. Their initial response to malicious comments consistently involves immediate, overt, and confrontational emotional expression. The experiences of these participants not only highlight the time-compressing effect of social media on emotional articulation but also reveal a related paradox: the instantaneous convenience of emotional catharsis on social media does not resolve negative feelings; instead, it exacerbates and disseminates them, and confrontational counter-responses prove ineffective in the long term. Collectively, the immediacy of social media lowers the threshold for emotional expression, rendering it an efficient space for catharsis. However, this venting serves only to rapidly displace emotions rather than to process and release them. Once individuals translate negative emotions into public textual statements within an uncertain public sphere, these emotions undergo misinterpretation, amplification, and counter-attack, ultimately returning to the expresser in a more intensified form.

“*When I saw nasty malicious comments, my first reaction was extremely impulsive; I immediately replied in the comment section to rebut, trying to clarify for the blogger and call out those who were speaking irresponsibly.”* (ST33).

“*My first instinct was to rebut each point, clarify my position, and prove there was no ill intent. I even carefully edited a lengthy reply to explain the relevant expertise. Yet after posting, it only attracted more malicious attacks, and the comment section became increasingly chaotic*. (ST27).

“*At the start, whoever commented, I had to fight back.”* (ST19)

#### 4.3.2 Continued exposure drives negative interactions to transform into cumulative psychological burden

##### 4.3.2.1 Emotional fluctuations and accumulation

This study found that negative online interactions have a significant, immediate, and sustained impact on the emotional states of university students. Participants generally reported that such interactions readily induce persistent negative emotional responses, including tension, anxiety, and unease, and in severe cases, can lead to somatic symptoms and substantial impairment of daily functioning.

On the psychological–emotional level, the sudden onset of negative online interactions typically triggers cognitive freezing and compound negative emotions (ST01). The term “stunned” denotes a temporary halt in cognitive processing, while “wronged and angry” constitutes a compound negative emotional state.

“*At first, I was completely stunned; then I felt both wronged and angry. I was just having a normal discussion, and suddenly I was being attacked by a group of people. I was extremely anxious, couldn’t even finish my meal, and that awful feeling lingered for most of the day—I couldn’t focus on studying at all*” (ST01).

At the somatic–behavioral level, the emotional responses of some participants had already transcended the psychological boundary, manifesting in physiological and action domains (ST04). First, the somatic responses (trembling, accelerated heart rate, heightened sensory sensitivity) indicate that the emotional intensity had exceeded the threshold of ordinary “bad mood” and entered a pathological state of physiological arousal. Second, action inhibition (fingers hovering, inability to proceed) reflects an individual’s loss of control driven by intense fear and uncertainty. Third, sensory overload (ringing in the ears) suggests that negative emotions rendered the individual abnormally sensitive to external stimuli.

“*I was trembling physically, my fingers hovering over the screen, frozen there for a long time, afraid to tap. My heart was pounding wildly, and I could even hear a ringing in my ears*” (ST04).

On the functional–temporal level, the impact of negative emotions did not cease with the end of the interaction but continued to permeate the individual’s daily functioning, disrupting core routine activities (ST08). The findings indicate that the emotional consequences of negative online interactions for university students are not only immediate but also persistent and cross-contextually pervasive, capable of producing both instantaneous effects and long-term distress, extending from psychological states to physiological responses and academic functioning.

“*This emotional state lasted about two or three days. Although I forced myself to sleep that night, those aggressive words kept replaying in my mind. I tossed and turned, unable to fall asleep, and the next day I could not concentrate in class at all”* (ST08).

##### 4.3.2.2 Social evaluation pressure

University students widely experience significant social evaluation pressure within negative online interaction contexts. This pressure manifests primarily as an excessive preoccupation with others’ perceptions and persistent apprehension regarding their own actions potentially eliciting negative appraisals. For university students navigating a critical period of identity formation and social relationship construction, online social platforms amplify the visibility and persistence of evaluations, rendering naturally spontaneous self-expression cautious and anxiety-laden. Some participants reported initially perceiving social platforms like “Moments” as safe spaces for acquaintances, conducive to freely sharing emotions and life achievements. However, they gradually recognized that casual expression could incur uncontrollable social evaluation (ST14), and even trust-based self-disclosure might transform into a psychological burden due to negative feedback. Similarly, other participants (ST24) exhibited heightened social sensitivity (ST28) during emotional expressions or opinion debates. Through these illustrative cases, university students aspire to authentically express emotions, stances, and achievements online. Nevertheless, their heightened sensitivity to evaluation inhibits the spontaneity of expression, even leading to enduring emotional distress following negative evaluations.

“*I used to think that Moments were for close friends, where I could freely share my joys, sorrows, and life accomplishments, and I also cared a lot about others’ evaluations—a single negative comment could affect me for a long time*” (ST14)

“*I always post updates with personal emotions and care a lot about others’ opinions*” (ST24)

“*I am quite impulsive by nature—when I encounter differing opinions, I can’t help but argue, and I am especially concerned about how others perceive me*” (ST28)

##### 4.3.2.3 Psychological burden and cognitive uncertainty

In the context of persistent negative online interactions, the social evaluation pressure experienced by university students frequently escalates into an accumulation of psychological burden. This manifests primarily as sustained attention to comment content and repetitive rumination on interaction processes. A subset of participants exhibited highly frequent comment monitoring behaviors (ST08). This recurrent checking was not driven by a need for information acquisition but rather by vigilance and anxiety regarding potential negative evaluations. The accompanying physiological arousal, such as an accelerated heart rate, indicates that the psychological burden has begun to manifest somatically. A more typical cumulative manifestation involves the temporal persistence of emotional and cognitive distress (ST29). When this burden remains unmitigated over time, individuals often enter a state of exhaustion characterized by the coexistence of avoidance and resistance (ST31). The cumulative psychological burden induced by negative online interactions, when severe, is sufficient to disrupt students’ academic pursuits, social engagement, and sense of self-efficacy.

“*I couldn’t help checking my phone every few minutes to see if there were new replies or private messages, and my heart rate noticeably increased.*” (ST08)

“*This feeling of irritation and grievance lasted for most of the day; I couldn’t help repeatedly recalling those words during class and even started to doubt whether I was really the problem. I was very anxious and couldn’t concentrate on listening.*” (ST29)

“*I was in a particularly bad state that day, unable to focus on anything else. I couldn’t stop repeatedly looking at the negative comments, and the more I read, the more annoyed I became—to the point where I felt resistant to posting anything at all.*” (ST31)

#### 4.3.3 Students regain a sense of control over digital interactions through a progressive coping pathway

##### 4.3.3.1 Emotion regulation

In the context of negative online interactions, undergraduate students’ initial coping responses frequently exhibit a pronounced tendency toward immediate reactions. This inclination represents not a carefully reasoned strategic choice, but rather a rapid behavioral output under heightened emotional arousal, primarily manifesting as emotional catharsis and direct refutation of negative comments or aggressive discourse. From a behavioral perspective, the immediate reaction tendency is first evinced by a strong impulse to retort (ST16), wherein “getting emotional” signifies that the behavior is not based on a rational analysis of the comment’s content but rather an stress-induced response during a rapid escalation of emotions. “Retorting point by point” reflects a response behavior characterized by distinct directness, one-by-one engagement, and inflexibility, implying that the individual, in that specific situational context, did not consider strategic avoidance or selective disregard. Instead, each negative comment was perceived as a provocation requiring direct counteraction. This “responding to each comment” behavioral pattern, while potentially offering momentary cathartic satisfaction on an emotional level, also readily draws the individual into a broader vortex of contention (ST29). The experience, “initially retorted (typed a few sentences in response)—the other party escalated (became more aggressive)—the scope of the conflict expanded (pulled others in to agree),” corroborates this observation. The immediate reaction tendency of undergraduate students in the early stages of negative online interactions is a high-arousal, low-reflection coping mode. While it can swiftly release immediate discomfort, it markedly lacks strategic foresight and consideration of subsequent consequences.

“*At first, I got carried away by my emotions and rebutted each commentator one by one*” (ST16)

“*I initially typed a few rebuttals, but the other party only intensified their attacks and even enlisted others to join in*” (ST29)

##### 4.3.3.2 Emotional boundary regulation

Following an initial tendency toward immediate reactions in negative online interactions, some university students, as conflict experiences deepened and emotional arousal levels gradually subsided, began to exhibit more adaptive coping shifts. This transition involved a progressive move from impulsive emotional venting and direct rebuttal toward more considered adjustment strategies, reorienting coping efforts toward the management of attention, expression, and engagement. Behaviorally, these adjustment strategies first manifested as selective inattention and withdrawal of attention (ST10). This approach, characterized by “retaining expression while shielding feedback,” effectively established a balance between self-presentation and self-protection needs (ST02), representing a more thorough operation compared to a previous participant. Disabling comment sections served to block the generation of new negative feedback at its source, while setting a post to “visible to self only” allowed the expression to remain while withdrawing the content from public view. This indicates that university students, when confronted with persistent negative interactions, do not passively endure but learn to utilize social media privacy and permission settings to reconstruct their online boundaries, and the acquisition and application of these adjustment strategies reflect their evolving coping capabilities within the digital social environment.

“*Later, I gradually calmed down and did not continue replying, nor did I delete my own posts; I simply stopped looking at the related comments and private messages*” (ST10)

“*Later, I simply turned off the comment section and set that post to ‘only visible to myself,’ no longer wanting to see those negative evaluations*” (ST02)

##### 4.3.3.3 Emotional interaction management

Following an initial phase of emotional catharsis and limited attempts at coping strategies, a subset of university students, when confronted with escalating or recurrent negative online interactions, ultimately develop more thorough avoidance and withdrawal behaviors. This stage differs from the previously mentioned selective inattention, manifesting instead as the active severing of social contact, a complete retreat from public discourse, and even a holistic avoidance of online social spaces (ST01, ST09, ST12). This description fully illustrates the behavioral trajectory from “active coping” to “complete withdrawal”: “replying to comments one by one to explain”—“the more I argued, the more tired I became”—“I directly deleted the comments and never touched that post again.” Deleting comments signifies a complete negation of existing interactions (ST03). In contrast to the previous participant, the action of “setting the account to private” here signifies a comprehensive retreat from a “public or semi-public social status” into a private sphere “visible only to authorized individuals” (ST17). This is no longer merely an action taken toward a specific post or comment, but a structural adjustment to the overall accessibility of one’s online identity. After setting the account to private, although the individual has not completely left the platform, their degree of social exposure has significantly diminished.

*“Initially, I wanted to argue with them, replying to each comment to explain. However, as the arguments escalated and became more exhausting, I simply deleted the comments and never revisited that post.”* (ST01)

*“I immediately deleted that comment and set my account to private.”* (ST03)

*“I would discuss it with friends, and after each conversation, I felt more comfortable.”* (ST12)

*“Psychology classes at university have covered coping mechanisms for cyberbullying. I also seek consultation with a psychology counselor or can speak with my academic advisor, rather than internalizing these issues.”* (ST17)

*“I proactively discuss these matters with close classmates and friends, and also confide in my family about these concerns.”* (ST09)

#### 4.3.4 Resource mobilization facilitates adaptive adjustment in digital environments

##### 4.3.4.1 Digital literacy performance

In the digital era, core individual digital literacy resides in the critical appraisal of information and the accurate comprehension of interactive contexts, transcending mere technical proficiency. For university students confronting potentially adverse online interactions, digital literacy bifurcates into two principal dimensions: firstly, information discernment capability, which encompasses the ability to scrutinize and question the veracity and credibility of online content; and secondly, contextual understanding, referring to an individual’s capacity to interpret communication content logically by integrating the interactive background, relationship type, and underlying motivations, thereby mitigating misunderstandings and conflicts. During interviews, ST08 articulated that when processing potentially false or misleading information, they first assess its authenticity, demonstrating an awareness of proactive verification rather than passive reception. Teacher TT01’s observations of students yielded a comparable conclusion, substantiating that a portion of university students, when faced with negative interactions, can mobilize cognitive resources to judiciously evaluate information sources and content, exhibiting a relatively mature capacity for digital judgment. The pivotal role of digital literacy in negative online interactions extends beyond averting emotional responses; it empowers individuals to identify potentially misleading or aggressive content through rational assessment and contextual comprehension, facilitating more appropriate responses.

“*I try to judge the truthfulness of what the other person is saying*” (ST08)

“*Some students are more rational; they first assess whether the information is true or false*” (TT03)

##### 4.3.4.2 Psychological resilience

In addressing negative online interactions, individual traits, particularly expressive tendencies and personality characteristics, are significant internal factors influencing the behavioral choices of university students. Compared to students who are introverted and tend to avoid conflict, extroverted individuals are more likely to assert their positions in negative situations and exhibit greater proactivity in their responses (ST02). This observation indicates that an extroverted personality is often associated with positive interpersonal relationships and stronger environmental adaptability, leading individuals to favor active communication over passive avoidance when confronted with online conflict (ST06). Furthermore, personality traits are not formed in isolation but are closely linked to early familial upbringing experiences and robust social support systems. Emotional support from family and friends enhances an individual’s psychological security and provides an emotional foundation for maintaining confidence and expressing oneself rationally during negative interactions. Consequently, university students with extroverted personalities and strong interpersonal relationships are more inclined to adopt proactive and constructive coping strategies in negative online interactions, thereby mitigating the adverse psychological impact of conflict.

“*I believe these students are usually lively and outgoing in personality; their offline interpersonal relationships are also good, and they possess stronger personal adaptability.*” (TT02)

“*These students tend to have a more cheerful personality; their family upbringing is better, and they receive more love from parents and friends.*” (TT06)

##### 4.3.4.3 Social support resources

Social support resources play a crucial buffering and regulatory role in navigating negative online interactions. Extensive and stable interpersonal networks, along with diverse and accessible support channels, can furnish individuals with essential resources, such as emotional outlets, thereby mitigating the psychological impact of adverse interactions (TT04). This underscores the significance of real-world support systems as a primary source for maintaining equanimity in virtual environments. When students possess dependable confidants, they are less prone to succumbing to panic when confronted with online aggression or misinterpretation (TT08). The efficacy of social support resources is contingent not only on the support network itself but also on individual psychological maturity and relational trust. Psychologically mature students are more inclined to actively seek assistance, and robust interpersonal relationships facilitate the activation of support resources. Such students are also more proactive in constructing and maintaining their support networks.

“*Students who exhibit greater composure when confronted with negative online interactions possess discernible characteristics, including robust real-world social support networks characterized by stable friendships and reliable confidants among family or instructors.*” (TT04)

*“These students are psychologically mature, have good interpersonal relationships, and trust teachers, classmates, and are outgoing.”* (TT08)

## 5 Discussion

### 5.1 Psychological dynamics in technology-mediated social interactions

This study indicates that the psychological experiences of university students during negative online social interactions are not merely instantaneous reactions to single events, but rather a dynamic process of continuous accumulation, adjustment, and reconstruction within a perpetually connected digital environment. This finding primarily stems from Theme 1, “digital platform conductivity shapes the emergence of negative online social interactions,” and Theme 2, “continued exposure drives negative interactions to transform into cumulative psychological burden,” presented in the results section. The research reveals that characteristics of digital platforms such as open dissemination, instant connectivity, the persistence of interaction traces, and high visibility do not solely serve as the external context for negative interactions; rather, they profoundly alter how individuals experience and comprehend interactive events. Compared to traditional face-to-face social interactions (Pathak et al., 2023; Rachadell, 2022), the technologically mediated environment facilitates the rapid diffusion, persistent retention, and repeated access of interaction information. This compels individuals to contend not only with the interaction itself but also with the compounded pressures arising from the platform environment, social evaluations, and relational networks (Nesi et al., 2018b). Consequently, the psychological repercussions of negative online interactions are not confined to transient emotional responses during the event (Khosdelazad et al., 2020) but represent a process of gradually accumulating psychological burdens through continuous exposure and ongoing interpretation (Kunst and Mesoudi, 2025), which subsequently influences subsequent behavioral adjustments and modes of social engagement. This finding expands upon existing research that predominantly focuses on the relationship between negative online interactions and mental health outcomes such as depression (Mayer et al., 2025) and anxiety (Anto et al., 2023; Keles et al., 2020) by shifting the analytical focus from “interaction outcomes” to “interaction processes.” It underscores that psychological experiences are dynamically formed, accumulated, and evolved within the ongoing process of social interaction supported by technological mediation (Kunst and Mesoudi, 2025). This discovery enriches the understanding of technologically mediated social interactions in cyberpsychology and demonstrates that digital platforms have become significant social spaces influencing university students’ psychological experiences and the construction of social relationships.

### 5.2 Adaptive coping in dynamic processes

Existing research typically frames coping strategies as behavioral choices individuals make after experiencing online stressors, often categorizing them as either active or avoidant coping (Wolfers and Utz, 2022). However, this study further reveals that university students’ responses to negative online social interactions are not merely strategic selections but rather a dynamic, adaptive process characterized by continuous adjustment in response to evolving interaction contexts. This finding primarily emerges from Theme 3, “students regain a sense of control over digital interactions through a progressive coping pathway.” The results indicate that students confronting negative online interactions generally progress through a series of stages: initial emotional reactions, interpretation of the interaction’s meaning, and subsequent behavioral adjustments and digital boundary renegotiation. Some students initially mitigate psychological uncertainty by expressing emotions, engaging in communicative interpretation, or seeking confirmation. When negative interactions persist or recur, they gradually shift towards reducing interaction exposure, modifying platform usage patterns, and redefining their personal digital spaces. This suggests that the coping process is not a fixed behavior for a single event but rather an iterative evolution within ongoing interactions, involving cognitive appraisal, emotional regulation, and social relationship negotiation.

This process also highlights the dynamic nature of adaptive coping in digital environments, distinguishing it from traditional stress contexts (Smith, 2024). Traditional stress coping theories predominantly focus on individuals’ recovery processes following a stressful event. In contrast, the continuously connected nature of digital social interactions subjects individuals to environments where re-exposure and reinterpretation of interaction meanings are constant possibilities. As revealed in themes 1 and 2, the high transitivity and persistence of interaction traces on digital platforms can continually reawaken negative experiences and accumulate psychological burdens. Consequently, students must continuously adjust their interaction strategies to maintain psychological security and social engagement. Within this process, coping is not simply about disengagement or avoidance but rather a continuous effort to balance digital social participation with the mitigation of psychological risks (Compas et al., 2001; Valkenburg et al., 2022). Therefore, this study also expands the explanatory scope of stress coping research in digital environments (Zhu and Shen, 2020; Zhao et al., 2025), emphasizing that university students’ digital adaptive capacity is not only reflected in their resilience after negative interactions but more significantly in their ability to dynamically adjust cognitive processes, behaviors, and interaction boundaries within a continuously connected environment.

### 5.3 Resource mobilization in digitally connected environments

This study found that university students do not passively experience psychological distress from negative online social interactions; they actively mobilize various personal and social resources throughout ongoing interactions to achieve psychological adjustment and adaptation within digital environments. This finding, primarily derived from Theme 4, “Resource mobilization facilitates adaptive adjustment in digital environments,” further reveals that the process of digital adaptation is not contingent upon a single psychological capacity but rather is a dynamic process involving the joint contribution of multiple resources, including digital literacy, emotion regulation, social support, and psychological resilience.

The research results indicate that different resources play complementary roles at various stages of negative online interactions. First, digital literacy assists students in comprehending the operational logic of digital platforms, identifying interactional risks, and forming more rational judgments about interactions, thereby reducing uncertainty stemming from misunderstandings or conflicts (Koltay, 2011). Second, emotion regulation enables students to modulate affective responses after encountering hostile comments, relational conflicts, or negative evaluations, thus preventing immediate emotional experiences from further impacting subsequent social participation. Third, social support provides students with crucial channels for emotional expression, meaning reconstruction, and action advice (Wesley and Booker, 2021). The findings demonstrate that when students possess stable networks of support from friends, family, or teachers, they are more likely to reinterpret negative interactional experiences and restore trust in digital social relationships. Finally, psychological resilience encourages students to gradually resume positive engagement within continuous interactional contexts, achieving long-term adaptation by adjusting interactional strategies and re-establishing digital boundaries (Crowder et al., 2026).

This resource integration process aligns with the incremental coping pathways revealed in Theme 3. Students’ behavioral adjustments do not solely originate from internal psychological capacities but are gradually formed through the synergistic effect of digital competencies, emotion management, and social relationship support. For instance, some students mitigate the impact of negative interactions by reducing unproductive exchanges, modifying platform usage patterns, and seeking real-world support, a process that exemplifies the dynamic synergy between personal and social resources. Consequently, unlike existing research that focuses on the independent roles of digital literacy (Koltay, 2011), social support (Wesley and Booker, 2021), or psychological resilience (Crowder et al., 2026), this study further elucidates how these resources are embedded within authentic digital interaction processes and jointly facilitate an individual’s transition from negative experiences and behavioral adjustments to sustained adaptation. Psychological adaptation in digital environments is not the outcome of a single psychological capacity but rather a process of continuous integration of personal competencies, social relationships, and situational resources within technologically mediated social interactions. This finding expands the understanding of digital adaptation in online psychology, emphasizing that comprehending the digital well-being of university students requires not only examining the types of negative interactions they encounter but also further investigating how they utilize diverse resources to maintain psychological safety and social participation in persistently connected digital environments.

### 5.4 Implications for cyberpsychology and digital citizenship

This study advances the understanding of technologically mediated social interaction within cyberpsychology from both process and contextual perspectives, offering practical implications for digital citizenship education in higher education institutions. Firstly, it supplements existing research, which predominantly focuses on the relationship between cyberbullying (John et al., 2018; Kwan et al., 2020) or cyberviolence (Antoci et al., 2016) and mental health outcomes, by shifting the emphasis to the processes of psychological experience formation and behavioral adjustment within the daily digital social interactions of university students. The findings indicate that negative online social interactions are not confined to a minority of extreme online harm incidents but are embedded within daily digital life, including course-related communication, peer interactions, interest-based communities, and social media expression. Specifically, Themes 1 and 2 demonstrate that the high transmissibility, interactive persistence, and informational visibility of digital platforms allow negative interactions to be continuously experienced, interpreted, and accumulated, thereby influencing students’ subsequent psychological states and modes of social engagement. Secondly, this research offers a process-oriented explanation for psychological adaptation in digital environments (Fortuna, 2023). Diverging from a view of coping as a singular behavioral choice made after confronting online stressors, this study, integrating Themes 3 and 4, reveals that students’ adaptation processes manifest as a continuous evolutionary trajectory encompassing emotional regulation, reconstruction of interaction meaning, adjustment of digital boundaries, and resource integration. This finding does not propose a novel theory of psychological adaptation but rather provides a contextualized augmentation to existing research on stress coping, digital literacy, and social support. Further elucidating how individuals maintain psychological safety and social engagement capabilities within persistently connected digital environments.

On a practical level, this study holds implications for digital citizenship education and student psychological support in higher education. Cultivation of digital literacy in universities should not solely focus on online safety knowledge and technological proficiency but should also enhance capacities in digital communication, emotional regulation, conflict management, and healthy interaction. Concurrently, student psychological support systems need to address the cumulative psychological stress arising from routine digital interactions, rather than being exclusively centered on extreme events such as cyberbullying (John et al., 2018; Kwan et al., 2020) or cyberviolence (Antoci et al., 2016). Through psychological support (Rivera et al., 2025) and social support (Wesley and Booker, 2021), models for guiding digital interactions and mediating conflicts can be established, enabling higher education institutions to assist students in developing more positive, responsible, and sustainable modes of digital engagement.

### 5.5 Limitations and future research directions

This study explores university students’ negative online social interaction experiences and psychological adaptation processes in higher education digital environments from a cyberpsychological perspective. It provides process-oriented insights into meaning construction, behavioral adjustment, and resource mobilization in technology-mediated interactions among young adults. However, several limitations should be acknowledged.

Firstly, the findings primarily reflect the digital interaction experiences of university students within a specific higher education context, as they are based on interview data from students at a university in Jiangsu Province, China, and teachers with observational experience of digital interactions in educational settings. Thus, the study does not aim to explain the psychological processes of online interactions across all populations and social contexts. Variations in cultural backgrounds, university types, platform ecosystems, and digital interaction norms can influence students’ understanding, evaluation, and coping strategies for negative online interactions. Future research could expand to diverse regions, university types, and cross-cultural contexts to compare commonalities and situational differences in students’ digital interaction adaptation processes.

Secondly, teacher participants served primarily as providers of contextual information regarding interaction practices within the university’s digital community, rather than as representatives of students’ private online experiences. Given that teachers typically have more exposure to course collaboration, student organizations, and education-related digital communication, and less insight into students’ private social interactions, recreational activities, and informal online behaviors, their perspectives are mainly used to supplement the understanding of the university’s digital interaction environment and cannot substitute for students’ own experiences. Future research could incorporate student digital diaries, online ethnography, platform interaction analysis, or multi-stakeholder interviews to understand interaction experiences across different digital scenarios from a more diverse range of perspectives.

Thirdly, this study employed cross-sectional qualitative interviews, relying primarily on participants’ recollections of past digital interaction experiences. While this approach can reveal subjective experiences and meaning construction in depth, it is challenging to fully capture the real-time dynamics of psychological changes throughout continuous interaction processes. Considering the instantaneous and evolving nature of digital social interactions, research could integrate longitudinal interviews, digital diaries, or mixed-methods designs to track students’ digital interaction experiences, psychological adjustments, and behavioral adaptation processes.

Finally, this study focused on how university students adapt through resources such as digital literacy (Koltay, 2011), emotion regulation, social support (Wesley and Booker, 2021), and psychological resilience (Odgers and Jensen, 2020). However, it did not further compare the differential effects of various resource combinations, platform characteristics, and interaction contexts. Future research could combine qualitative and quantitative methods to further analyze how different digital platforms, interaction types, and individual resource configurations jointly influence psychological dynamics and behavioral adjustments. This would advance cyberpsychology research from describing digital adaptation processes to explaining the conditions for positive digital adaptation, thereby providing theoretical underpinnings for digital citizenship education in higher education, student psychological support, and the development of healthy digital environments.

## 6 Conclusion

As digital media become increasingly integrated into university students’ learning, daily lives, and social interactions, negative online social interactions have emerged as important experiences within higher education digital environments. Drawing on a cyberpsychological perspective, this study examined the psychological dynamics and adaptation processes underlying university students’ experiences of negative online social interactions. The findings indicate that such interactions are not isolated conflict events but dynamic processes shaped by continuously connected digital environments. Specifically, the high conductivity of digital platforms creates conditions for negative interactions, continuous exposure contributes to the accumulation of psychological burdens, and students gradually achieve adaptation through progressive coping pathways and the integration of digital literacy, emotional regulation, social support, and psychological resilience. These findings suggest that students’ psychological experiences in digital environments develop through ongoing processes of meaning construction, behavioral adjustment, and resource mobilization rather than through simple stimulus–response patterns.

This study extends existing research beyond the psychological consequences of negative online interactions to their formation and evolution processes. It highlights that students’ negative online experiences should be understood as part of everyday technology-mediated social interactions rather than being limited to extreme cases such as cyberbullying or online violence. By capturing students’ lived digital experiences, this study provides a process-oriented perspective on how young adults interpret negative interactions, adjust behavioral boundaries, and maintain social participation in connected digital environments.

Practically, the findings provide implications for university digital citizenship education and student psychological support. Universities should move beyond emphasizing online safety and technical skills by also strengthening students’ digital communication abilities, emotional regulation, and awareness of healthy online interactions. Psychological support systems should recognize the cumulative pressures arising from everyday digital interactions rather than focusing only on explicit online harms. In addition, university digital platforms and online communities should develop clearer interaction norms, conflict management mechanisms, and support functions to foster safer, more inclusive, and psychologically supportive digital environments.

Overall, understanding university students’ negative online social interactions requires attention not only to potential psychological risks but also to how individuals construct meanings, adjust strategies, and achieve adaptation within continuously connected digital environments. This process-oriented perspective advances understanding of digital social interactions in higher education and provides new directions for future research on youth digital well-being, digital citizenship education, and healthy digital environments.

## Data Availability

The raw data supporting the conclusions of this article will be made available by the authors, without undue reservation.

## Appendix 1

### Student interview questions (simplified summary)

1. Can you recall a particularly memorable online interaction? What happened? On which platform? Why did you participate or get involved?
2. What was your first reaction? Did you experience any significant emotions, such as anger, resentment, or anxiety? How long did these feelings last? Did they affect your state of mind that day?
3. How did you interpret the other person’s words or actions? Did you consider why they expressed themselves that way? Did you assess the authenticity or background of the information?
4. What did you specifically do at the time? Did you change your approach later? Did you try replying, deleting, ignoring, or confiding in someone else? Was there a turning point in this process? Why did you change your approach later?
5. After experiencing similar situations several times, do you feel that you have changed? Now, if you encounter a similar situation, will your approach be different than before? Do you feel a gradual adaptation?
6. Do you discuss these things with classmates or friends? Do their opinions influence you? Did any teachers or school staff suggest you seek help?
7. Looking back now, what does that experience mean to you? Did it change your views on online expression? If you were to give advice to someone, what would you say?

### Teacher interview questions (simplified summary)

1. Have you noticed any changes in students’ online interactions? Have any students shown emotional fluctuations due to online events?
2. Could you share a student case that has left a deep impression on you? What was the student’s state at the time?
3. From your observations, what are the typical emotional reactions of students? Are there differences in these reactions among different students?
4. How have you observed students generally handling these situations? Are there any particularly mature or unique coping mechanisms?
5. Have you noticed that some students appear more composed when facing similar situations? What are the typical characteristics of these students?
6. When students encounter these kinds of problems, how do you usually communicate with them? In your opinion, what kind of support is most helpful to students?
7. How do you understand the current situation of university students in the online environment? What do you think they need most?
